# Lingonberry (*Vaccinium vitis-idaea* L.) Fruit Phenolic Bioactivities—A Review of In Vitro and In Vivo Human Studies

**DOI:** 10.3390/microorganisms12091850

**Published:** 2024-09-06

**Authors:** Pirjo Pärnänen, Sari Niikko, Hanna Lähteenmäki, Ismo T. Räisänen, Taina Tervahartiala, Timo Sorsa, Annamari Ranki

**Affiliations:** 1 Department of Oral and Maxillofacial Diseases, Head and Neck Center, Faculty of Medicine, University of Helsinki and Helsinki University Hospital, 00290 Helsinki, Finland; 2 Dental Clinic Miun Paikka, 80100 Joensuu, Finland; 3 Dental Clinic Kruunu, 33200 Tampere, Finland; 4 Division of Oral Diseases, Department of Dental Medicine, Karolinska Institutet, 17177 Stockholm, Sweden; 5 Department of Dermatology, Allergology, and Venereology, Inflammation Center, University of Helsinki and Helsinki University Central Hospital, 00250 Helsinki, Finland

**Keywords:** lingonberries, *Vaccinium vitis-idaea* L., phenolics, human studies, bioactive molecules, fermentation, lactobacilli

## Abstract

This review is focused on the effects of lingonberry (*Vaccinium vitis-idaea* L.) fruit phenolic compounds in human in vitro cells and in vivo clinical studies. Studies with lingonberries, lingonberry juice/lingonberry nectar/fermented lingonberry juice, and phenolic fractions with active molecules are reviewed. Lingonberry’s bioactive substances have a diverse range of antimicrobial, anti-inflammatory, antiproteolytic, anticancer, and antioxidant properties. Fermentation of lingonberries and modulation of the dysbiotic microbiome to a more symbiotic composition by favoring the growth of lactobacilli and inhibiting the growth of human opportunistic pathogens are discussed. Research results suggest that more studies on humans are needed.

## 1. Introduction

Generalized resistance to antibiotics increases the pressure to find new, effective, and safe substances from natural plants. Natural substances and their potential molecules for the treatment of diseases can be effectively mapped with current techniques, and the most suitable ones can be selected for further development. Lingonberries (*Vaccinium vitis-idea* L.) have a versatile spectrum of antimicrobial, anti-inflammatory, antiproteolytic, anticarcinogenic, and antioxidant effects. The effects of lingonberries are based on the vitamins, minerals, fibers, and phenolic compounds they contain. Most of the lingonberry studies have been conducted with animal cells or animals, which only give indications of possible effects in humans. A problem in interpreting the research results of clinical human trials has been the fact that berry mixtures have often been used, and thus, it has been impossible to determine the effects of lingonberries alone. The interpretation of the results of the studies is complicated by the different isolation methods and concentrations of the phenolic substances in the berry fractions, which affect the activity. The composition of lingonberry fruits has been mapped in numerous studies [1,2,3]. The main groups of phenolic substances are flavonoids [anthocyanins, flavonols (quercetins), flavanols (catechins), and phenolic polymers (proanthocyanidins)], phenolic acids, lignans, and stilbenes (resveratrol). A total of 28 phenolic compounds have been isolated from lingonberry fruits, especially proanthocyanidin type-A dimers and trimers, anthocyanins (cyanidin-3-galactoside, cyanidin-3-arabinoside, cyanidin-3-glucoside), quercetin glycosides, and catechins [1,2]. In addition, lingonberries contain, e.g., A, C, and E vitamins and minerals that participate in antioxidant reactions, as well as plenty of natural sugars (mainly glucose, fructose, and sucrose), 8.2 g/100 g. The profile of lingonberry anthocyanins differs from its close relatives, such as the blueberry (*Vaccinium myrtillus*) and cranberry (*Vaccinium oxycoccus* L., *Vaccinium macrocarpon* Ait.) compositions. Lingonberries contain appr. 10,000 compounds, of which appr. 1000 are characteristic of lingonberries [4]. Processing of lingonberries, for example, by heating, enzymatic treatment, storing, or by different extraction methods of the fractions, causes changes in phenolic composition, which can reduce bioactivity. The phenolic compounds of the lingonberries are fermented by intestinal bacteria (possible prebiotic effect) and excreted in the urine as metabolites. The cyanidin-3-galactoside and its metabolites from whole lingonberry berries are excreted in the urine within 4–12 h [5]. Polyphenols isolated from lingonberries by extraction are metabolized in the large intestine by intestinal bacteria but retain their activity [6]. According to a study with CaCo-2 cells, the ethanol extract of the lingonberry berries does not significantly affect the absorption of well-absorbed drugs [7]. Phenolic substances have been found to have a protective effect against many aging-related diseases, such as cardiovascular diseases, asthma, damage caused by UV radiation, overweight, diabetes, and neurodegeneration [8]. Wild berries have their characteristic polyphenol composition [9]. Phenolics neutralize oxygen radicals, and eating enough vegetables and fruits has been found to prevent the occurrence of aging diseases such as cardiovascular diseases and cancer by preventing the oxidation of DNA, proteins, and lipids in the body [10]. Proanthocyanidins, anthocyanins, catechins, and quercetins play an important role in protecting cells from damage caused by oxidation.

This review presents the effects and dosing of phenolic compounds from lingonberry fruits, fractions isolated from them, lingonberry juice, lingonberry nectar, lingonberry puree, and fermented lingonberry juice based on the research data accumulated so far on humans, human cells, and human opportunistic pathogenic microbes. Web of Science/Pubmed database searches have been made, excluding studies with animals, lingonberry leaves, or berry mixtures.

## 2. In Vitro Studies

### 2.1. Antioxidant and Cancer Cell Studies

Lingonberry catechins and proanthocyanidins inhibit the oxidation of human LDL in vitro [11]. In another study, lingonberry fruits were lyophilized, homogenized, and extracted with 0.75% (*v*/*v*) acetic acid, and anthocyanin, copigment, and non-anthocyanin polyphenol fractions were isolated. All fractions prevented reactive oxygen species formation in HepG2 cells [12]. Lingonberry cyanidin-3-galactoside is the most abundant anthocyanin in lingonberry fruits, which acts as the main antioxidant. The water extract from homogenized lingonberries has been found to inhibit the growth of human leukemia cells HL60 by inducing apoptosis, partly based on the antioxidant effect [13]. Fermented lingonberry juice inhibits the proliferation of aggressive human tongue epithelial carcinoma cells HSC-3 and SCC-25 and the invasion of HSC-3 by concentrations > 2.5 mg/mL [14]. Lingonberry lyophilized ethanol extract (anthocyanin fraction) has a significant inhibitory effect on the proliferation of human estrogen receptor-positive breast cancer cells (MCF-7, ATCC) and human colon cancer cells (HT29, ATCC) at 0.01–350 µg/mL and total extract 0.025–0.5% and 0.05–0.5% in the order mentioned above [15]. The proliferation of human cervical cancer cells (HeLa) and human colon cancer cells (CaCo-2) is inhibited by proanthocyanidins type A and B (median effective concentration 28.7 µg/mL) from homogenized berries’ tannin fraction with sugars, organic acids, carotenoids, and vitamin C removed [16]. Lingonberry (homogenized fruits with 80% acetone extraction) fraction inhibits the proliferation of HT-29 (EC_50_ < 40 mg/mL) and hepatocellular carcinoma cells HEPG2 (EC_50_ ≤ 20 mg/mL) [17]. The proanthocyanidin fraction of lingonberry fruits induces apoptosis of SW480 (primary) and SW620 (metastatic) colon cancer cells, EC_50_ 45 µg/mL and 35 µg/mL, respectively [18]. Human malignant melanoma (IGR39), colon adenocarcinoma (HT-29), and human renal cell carcinoma (CaKi-1) cells lose their viability with polymeric and mainly A-type dimers with proanthocyanidins and quercetin (EC_50_ < 200 µM). In this study, lyophilized and homogenized fruits were extracted with 70% aqueous acetone [19].

### 2.2. Effects on the Liver, Chronic Low-Grade Inflammation, Overweight and Diabetes Risk

Liver α-amylase and α-glucosidase are inhibited, and liver glucose uptake is improved by lingonberry extract in human HepG2 cells. Frozen berries were homogenized and extracted with hexane and 80% methanol. Benzoic acid derivatives, anthocyanins, and flavonols need further investigation to determine their potential role in glucose uptake [20]. This may be important in preventing overweight and type 2 diabetes mellitus. Lyophilized fruits were homogenized and extracted with 0.75% (*v*/*v*) acetic acid to isolate polyphenol and anthocyanin fractions. These fractions prevent inflammation caused by obesity-related hypertrophic fat cells and endothelial cell dysfunction in human 3T3-L1 fat cells and HUVEC placental endothelial cells [21]. Advanced glycosylation end products, AGEs, cause complications in diabetic patients. Lyophilized and homogenized lingonberry fruits extracted with 80% ethanol have been shown to have dose-dependent antiglycation effects with an IC_50_ of 13.5 μg/mL, and the active molecules are suggested to be quercetin-3-O-galactoside and cyanidin-3-O-glucoside [22]. Lingonberry extract inhibits the pancreatic digestive enzyme lipase, which breaks down triglycerides in the intestines, causing their absorption into the bloodstream to deteriorate. This prevents the development of excess weight. A total of 30 µM concentrations of proanthocyanidins, kaempferol, and resveratrol change the macrophages of fat cells into an anti-inflammatory form, reducing the expression of IL-6 and TNF-alpha cytokines, reducing the adverse effects of obesity-related low-grade inflammation, and activating the transcription factor STAT6, affecting gene expression [23].

### 2.3. Antimicrobial Effects

According to numerous studies, lingonberries have antimicrobial effects (bacteria, yeasts, and viruses as human pathogens). Most microbes can use their enzymes to modify the host’s proteins or structures or to secrete proteins that modulate the virulence of other microbes, influencing the host’s immunological response against pathogens. A dysbiotic microbiome can predispose to disease. Probiotics may prevent the course of neurodegenerative diseases such as Alzheimer’s and Parkinson’s diseases by improving the dysbiotic gut microbiome [24] and the symptoms of asthma, allergy, and chronic obstructive pulmonary disease (COPD) through immunomodulation [25].

Ground lyophilized lingonberries extracted with 70% methanol and sugars removed with solid-phase extraction (containing 23 mg/g anthocyanins, flavonols 10 mg/g, OH-cinnamates 6 mg/g, flavan-3-ols 6 mg/g, total phenolics 280 mg/g) inhibit the growth of Gram-negative bacteria *Salmonella enterica* and *Escherichia coli* strain-dependently at concentrations 0.8–7 mg/g by agar diffusion method and in liquid culture with 1 mg/mL. It is noteworthy that the growth of any of the tested *Lactobacillus* strains was not inhibited [26]. Lyophilized berries (acetone extracted and sugars removed) inhibited in liquid culture *Staphylococcus aureus* and *Salmonella enterica* sv. *Typhimurium* at 2 mg/mL concentration and phenolic extracts at 1 mg/mL. There was no inhibition of *Lactobacillus rhamnosus* [27]. Anthocyanin and anthocyanidin fractions from lingonberry juice concentrate (65° Brix fractioned by centrifugation and diluted to 5–50 µg/mL) inhibit *Neisseria meningitis*-adhesion of meningococcal bacteria to human HEC-1B epithelial cells [28]. Lingonberry juice fractioned by column elution with 70% ethanol (polyphenols 586.8 mg/g, flavanols 374.6 mg/g) inhibited biofilm formation of *S. mutans* at concentrations 0.5–1 mg/mL, *S. sobrinus,* and *S. sanguinis bacteria* with 0.5–2 mg/mL [29]. The effects of *Saccharomyces cerevisiae*-fermented lingonberry juice (FLJ, Lingora^®^, Berries United Ltd., Helsinki, Finland, phenolics 212 mg/100 mL, anthocyanins 7.1 mg/100 mL, sugars reduced to 3 g/100 mL) have been studied on oral microbes with disc diffusion method, including species *C. albicans*, *C. dubliniensis*, *C. glabrata*, *C. krusei*, and *C. tropicalis*, *Fusobacterium nucleatum*, *Aggregatibacter actinomycetemcomitans*, *P. gingivalis*, *S. mutans*, *S. sanguinis*, *S. salivarius* and lactobacilli. Over 10× concentrated FLJ inhibits the growth of these yeasts, periodontal pathogens, and streptococci, but not lactobacilli. Lactobacilli are considered to be probiotic and health-promoting. Laboratory experiments have shown the ability of *C. glabrata* cell wall-bound proteases to activate and process pro-matrix metalloprotease-8 (pro-MMP-8) to its active form (aMMP-8)—this MMP-8 activation is inhibited by FLJ [30]. Lingonberry phenolic fractions [total extract and anthocyanins] prepared from lyophilized berries with methanol and solid-phase extraction (sugars, organic acids, and amino acids removed) have also been found to have antiviral properties and inhibit the replication of influenza A (total extract, anthocyanin fraction) and Coxsackie B1 enteroviruses (total extract). The cytotoxicity of the fractions was tested with Madin-Darby Canine Kidney (MDCK) and Hep-2 cell lines. Both fractions had very low cytotoxicity, and the maximal tolerated concentration for the phenolic fraction was 3–10 g/mL and the 50% cytotoxic concentration (CC_50_) was 6–18 mg/mL. Anthocyanins were less toxic [31].

## 3. Clinical Human Trials

When consumed together with sucrose, a single dose of 150 g lingonberry purée or 300 mL lingonberry nectar (50% concentrated juice + 35 g saccharose), estimated anthocyanins 100 mg and 600 mg proanthocyanidins, prevented the rise in postprandial glucose and insulin levels and hypoglycemia, as well as the rise in free fatty acids [32]. The effects are due to the slower breakdown and absorption of sucrose. With light or dark bread, the glucose response was weaker [33]. This is thought to be due to the weaker inhibition of pancreatic alpha-amylase by phenols compared to alpha-glucose oxidase (specific for sucrose).

There is no clinical evidence of the effects of lingonberry in the prevention of urinary tract infection, but lingonberries contain more dimeric and trimeric proanthocyanidins of type A than cranberry [*V. oxycoccus* (European variety)] or *V. macrocarpon* [North American variety] [34]. These A-type proanthocyanidins are thought to be the most important inhibitors of bacterial adhesion.

### 3.1. Oral Effects

Dry mouth caused by drugs, cytostatic and radiation therapy, broad-spectrum antibiotics, aging, ill-fitting prostheses, or weakened immunity caused by disease exposes the oral microbiome to changes, such as yeast infection. Reduced salivation weakens the protective effect on the teeth, gums, and mucous membranes, which also changes the thickness and composition of the biofilm. Although natural berries are beneficial for health, in addition to active substances, they also contain a lot of natural sugars, such as glucose, fructose, and sucrose.

The effects of lingonberries in the mouth have been studied very little. There are six oral clinical studies on humans in the literature [35,36,37,38,39,40], one of which was conducted with lingonberry juice and five with FLJ.

Chronic mucocutaneous 2 ½-year remission of yeast symptoms in autoimmune polyendocrinopathy-candidiasis-ectodermal dystrophy (APECED, APS-1) patients with candidiasis without yeast medication was achieved using FLJ (Lingora^®^, Berries United, Helsinki, Finland; 0.1 g/mL, dw/v) as an oral rinse supplement (10 mL/day) in oral self-care [35]. The patient had suffered from recurrent oral, esophageal, gastric, vaginal, and anal candidiasis since childhood. The *Candida* species cultured and identified were *C. albicans*, *C. tropicalis* and *C. glabrata*. Pale, thick deposits on the mucous membrane of the mouth, which resembled a *Candida* infection, have been studied in infants under 12 months of age [36]. A total of 4 children out of 32 had mucosal changes, one of which was confirmed to have *C. parapsilosis* by cultivation. Lingonberry juice was compared to lemon juice and soda water administered as drops in the mouth when a change appeared, and follow-up took place after two weeks by calling nursing mothers from the clinic. It was found that acidity alone did not remove the appearance of oral thrush in three children, and their etiology remained unclear.

FLJ increases saliva secretion, lowers *Streptococcus mutans* and *Candida* yeasts, is prebiotic by increasing the number of lactic acid bacteria, lowers the risk of caries, and reduces the amount of plaque and gingivitis. It has a positive effect on the clinical parameters of periodontitis, lowering oral proteolytic burden, i.e., the levels of active matrix metalloprotease-8 (aMMP-8) in the oral fluid, as well as reducing the low-grade inflammation associated with periodontal diseases, and due to its safety, it is well suited to support oral self-care. FLJ used as an oral rinse enhances tooth brushing and maintenance of oral hygiene and contributes to the control of plaque [37,38,39,40,41]. FLJ oral rinse has been developed for safe oral use; the sugars have been reduced to a fraction using a patented method. Reducing sugars is essential because oral microbes such as *Candida* yeasts and streptococci use sugars in their metabolism.

### 3.2. Effects of Fermented Lingonberry Juice on Oral Microbes

**Figure 1:**
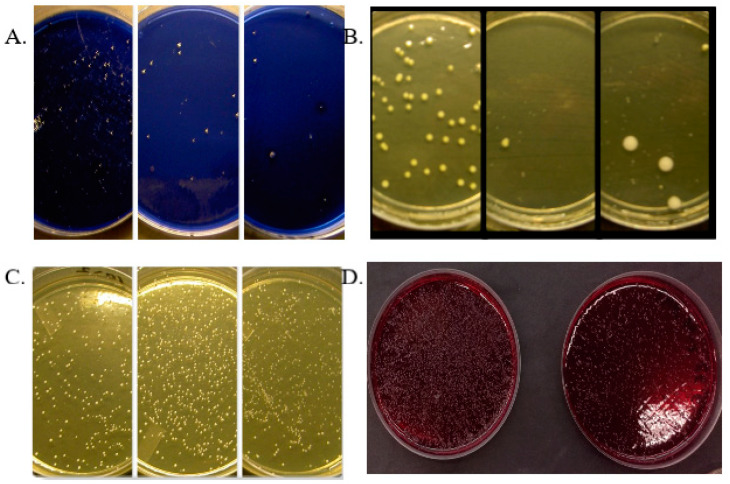
(**A**) *Streptococcus mutans* cultivation on MSB plate. Initial sample (left) and growth inhibition after 2 weeks of using fermented lingonberry mouthrinse (10 mL burst two times a day), and final sample 2 weeks after the end of use (right). (**B**) *Candida* cultures on SDA plates. Initial sample (left), growth inhibition after 2 weeks of using fermented lingonberry mouthrinse (middle, 10 mL ½ min 2 times a day), and final sample 2 weeks after the end of use (right). (**C**) Plating of lactobacilli. The initial sample (left) and the increase in growth after 2 weeks of using fermented lingonberry mouthrinse (10 mL ½ min burst 2 times a day), and the final sample 2 weeks after the end of use (right). (**D**) Growth of anaerobic oral bacteria on Brucella blood agar (initial sample on the left) and inhibition of growth after 2 weeks of lingonberry mouthrinse (10 mL mouthrinse ½ min 2 times a day, right image) [37].

Old age and mouth-drying drugs affect the number of oral microbes: a dry mouth predisposes to caries, which can be seen in the results of *S. mutans* smears as high values (Figure 1A). After using the FLJ oral rinse, the levels of *S. mutans* decreased significantly, and the effect remained even afterward (6 months) after the use of the FLJ oral rinse was stopped [39]. Oral *Candida*-yeast levels decreased significantly during the use of FLJ oral rinse (Figure 1B), and it is also effective against non-*Candida albicans* species such as *C. glabrata* and *C. parapsilosis*. The number of lactobacilli increased during the use of FLJ oral rinse (Figure 1C). The changes in the amounts of *S. mutans*, *Candida* yeasts, and lactobacilli remained long after the use of the mouthrinse was stopped [39]. The development of gingivitis, periodontitis, implant mucositis, and peri-implantitis is a multifaceted process in which self-care of the mouth and regular professional cleaning of the mouth are of central importance, especially in the prevention and maintenance of the diseases concerned. FLJ oral rinse significantly reduces periodontitis-related bleeding-on-probing (BOP) and visible plaque index (VPI) indices [37,39,40]. Figure 1D shows the clear growth-limiting effect of mouthrinse on anaerobic bacteria in a clinical trial [39].

**Figure 2:**
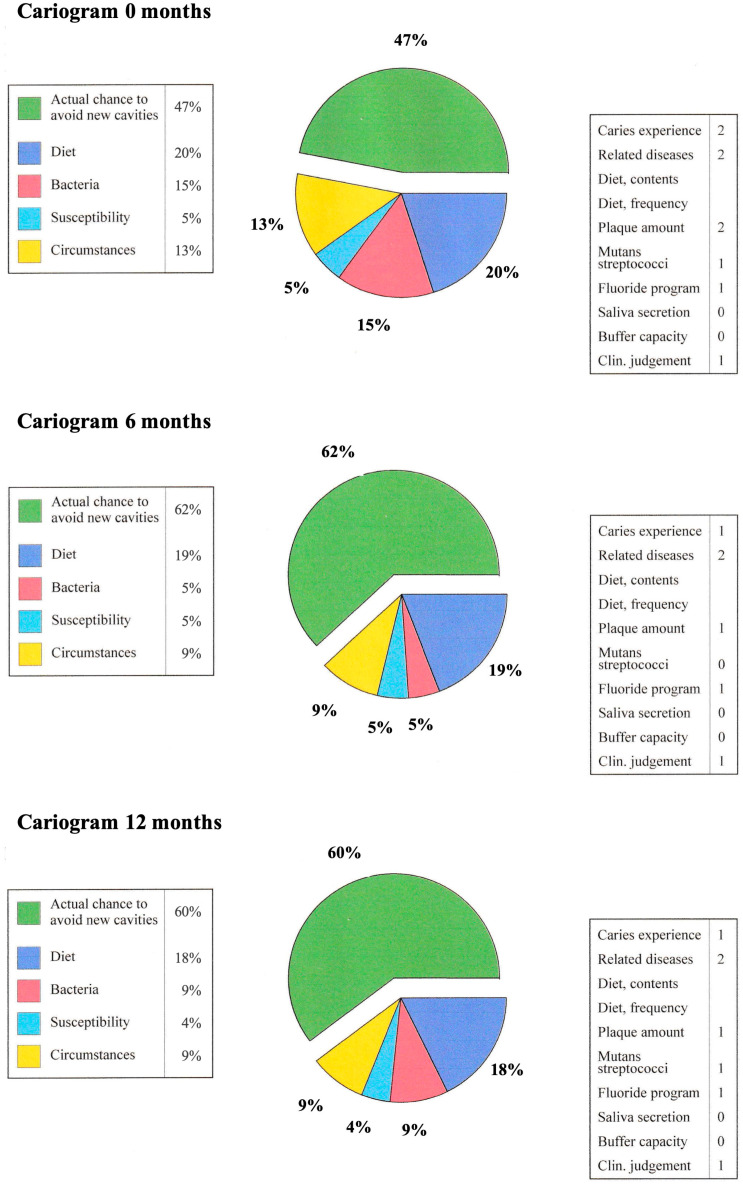
Cariograms of the participants (*n* = 25) in the study [41] at 0, 6, and 12 months. The average values of the oral variables have been used in the caries risk analysis of the Cariogram program [42] (the picture is a combination of the results given by the program at 0, 6, and 12 months). The green sector reflects the possibility of avoiding new caries. Dental fluoridation was performed at the reception at 0, 6, and 12 months. The participants were classified into a high-caries-risk group due to their high average age, medications, and illnesses. Their diet or oral self-care was not addressed in the studies.

FLJ oral rinse significantly increased saliva secretion and improved the buffer capacity of saliva but did not lower saliva pH or reduce the sensation of dry mouth [38]. Significantly reduced *Candida* yeasts and *Streptococcus mutans* bacteria, the amount of caries, low-grade inflammation of attachment tissues, gingival bleeding, and the amount of plaque also decreased significantly [37,39,40]. Caries risk can be assessed using a Cariogram analysis program (Cariogram, Version 3.00) [42]. The effect of different components on caries risk [39] can be seen in Figure 2. The average data of the patient material (*n* = 25) on different parameters from the time points 0, 6, and 12 months have been entered into the Cariogram program; the effect of the FLJ oral rinse can be seen in the reduction in caries risk (0–6 months) and the preservation of this caries protection for 6–12 months. Low-grade inflammation of the attachment tissues can be monitored by measuring the concentration of active matrix metalloprotease-8 (aMMP-8) in a mouthrinse sample. FLJ reduces low-grade inflammation and is anti-inflammatory [37,39,40]. aMMP-8 levels can also be measured when investigating the level of low-grade inflammation in common diseases, such as pre-diabetes screenings at the dentist’s office and COVID-19 as a risk disease [43].

## 4. Discussion

Phenolic bioactive molecules have been found in a variety of plants and food products, including green and black tea, coffee, red wine, fruits, berries, nuts, and seeds. They have cardioprotective, chemopreventive, anti-inflammatory, hepatoprotective, and antimicrobial effects. They also stabilize blood sugar levels and inhibit lipid peroxidation, and their total phenolic concentration correlates with antioxidant capacity [12,17]. Recent studies of berry bioactivities have reported anti-inflammatory gastrointestinal, antidiabetic, antiobesity, antihypertensive, immunomodulatory, anticarcinogenic, and neuroprotective effects [44]. Also, wild berries show similar bioactivities [45]. The phenolic composition varies between berries, and glycosylation, position, and number of hydroxyl groups in phenolic compounds affect the antioxidative capacity [46]. Some potential mechanisms for the health of commonly consumed berries have been revealed [47,48,49,50,51,52,53]. The bioavailability of phenolics is class-dependent, and several steps should be considered when evaluating what is the sufficient concentration of bioactive molecules reaching the target tissue to have health effects [54]. Most phenols are bound to food source components, and firstly, polymeric forms are broken down by gastric enzymes. The intestinal microbiome enzymatically converts phenols into metabolites, which are then absorbed, modified in the liver, enter plasma and target tissues, and finally are excreted into urine or feces. The intestinal metabolite spectrum, bioavailability, and bioactivity produced are microbial strain-dependent due to variations of enzymes in each species. These issues must be considered when planning future clinical trials.

Fermentation of food is considered beneficial for health. Fermentation of complex phenols to smaller metabolites is dependent on the microbial strain used. This enzymatic processing enables more efficient absorption from the gut and increases the bioavailability and bioactivity of phenolics [55]. The prebiotic inhibition by phenolics of the growth and adhesion of intestinal and oral opportunistic microbes in the gastrointestinal tract, transforming the microbiome into more symbiotic and favoring lactobacilli growth, is beneficial by decreasing low-grade inflammatory burden in the body. Lactobacilli also secrete antimicrobial peptides affecting other species, boost host immune defense, and help in maintaining mucosal barrier integrity. In addition, these transformed phenols inhibit pathogens and promote probiotic microbial growth and gastrointestinal health [55,56]. FLJ oral rinse, fermented with *Saccharomyces cerevisiae* yeast, has been shown to contain effective molecules and positive clinical outcomes from human in vivo studies [35,37,38,39,40]. New kinds of anthocyanin–epicatechin derivatives linked by ethyl bridges have been identified in lingonberries, blueberries, and cranberries [57]. These hybrid molecules are proposed to form during storage or fermentation in winemaking, are stable, and resist gastric digestion. The health effects of these molecules are unknown. Fermentation does not alter markedly total phenolic/anthocyanin content (fermented juice 2.12 mg/mL, 71 µg/mL; unfermented juice 2.7 mg/mL, 55.2 µg/mL [58]). Lingonberries contain 1.5× more phenolic compounds compared to cranberries [58]. The peel of lingonberry contains approximately the same amount of total phenolics but 100× less anthocyanins [23].

This review aims to give an overview of those studies published so far on the effects of lingonberry phenolic substances on humans in vitro and in vivo. The composition of lingonberry fruits has been studied extensively. The phenolic bioactive molecules have multiple beneficial effects on cell and clinical levels in humans. The effects seem to be concentration/tested human cell type or microbial strain dependent, often showing synergistic activity. Effective molecules are purified and identified in in vitro studies, but analyses are more complicated in in vivo human studies because of the interaction and metabolization of the molecules by host and microbial enzymes. The lack of human clinical studies is obvious, but promising results of active molecules (in parenthesis) from the in vitro and in vivo human studies carried out so far identified are anticancer (cyanidin-3-galactoside, anthocyanin, proanthocyanidins type A and B, quercetin), anti-inflammatory (polyphenol and anthocyanin fractions, proanthocyanidins, kaempferol and resveratrol), anti-proteolytic (anthocyanins, phenolics), antioxidant (catechins, proanthocyanidins, polyphenols), antimicrobial (polyphenols and anthocyanins), and metabolic (benzoic acid derivatives, anthocyanins, flavonols, quercetin-3-O-galactoside, cyanidin-3-O-glucoside). In vitro studies lay the groundwork for new targeted natural applications to be developed for cardiovascular diseases, metabolic syndrome, cancer prevention, microbial diseases, inflammatory diseases, and obesity. However, more clinical human studies are needed to provide evidence of the versatile, beneficial effects of lingonberry phenolic substances on health.
